# A minimally invasive approach with fertility preservation in a young woman with distinct bilateral ovarian masses: a case report and review of the literature

**DOI:** 10.5935/1518-0557.20140088

**Published:** 2014

**Authors:** Márcia M. Carneiro, Ivete de Ávila, Patrícia S. Gouvea, Maria das Graças M. Torres, Ivone D. S. Filogônio

**Affiliations:** 1 Department of Obstetrics and Gynecology, Universidade Federal de Minas Gerais, Brazil; 2 Biocor Hospital Belo Horizonte, Minas Gerais, Brazil

**Keywords:** adnexal masses, laparoscopy, ovarian cysts, dermoid cyst, fertility preservation

## Abstract

Adnexal masses are relatively common, contributing to gynecologist office volume and surgical case load. The development of minimally invasive techniques and a greater focus on fertility preservation have led to the favoring of a laparoscopic approach with ovarian cystectomy, when possible. We report the case of a young woman presenting with two simultaneous, distinct ovarian masses who was successfully treated by laparoscopy with preservation of both gonads. A minimally invasive surgical approach by laparoscopy with preservation of both ovaries is feasible and crucial, even in rare and difficult cases such as the case presented.

## INTRODUCTION

Adnexal masses are relatively common, accounting for many gynecologic consultations and contributing to surgical case load. Masses presenting during the reproductive years are nearly always gynecologic, and most are functional cysts. A contemporary preoperative workup in this setting involves history taking and physical examination; laboratory tests, including tumor markers such as cancer antigen 125 (CA-125); and imaging studies, usually including transvaginal ultrasound (TVUS) (ACOG, 2007). The development of minimally invasive techniques and a greater focus on fertility preservation have led to the favoring of a laparoscopic approach with ovarian cystectomy, when possible. Thus, laparoscopy has become the accepted approach, as it may help to avoid laparotomy for the treatment of benign ovarian disorders (Liberis *et al.*, 2009; Medeiros et al., 2009).

We aimed to report the case of a young woman presenting with simultaneous, distinct ovarian masses who was treated by laparoscopy with preservation of both gonads and to review the current literature on the subject. A review of the available literature was conducted by searching Medline and PubMed using the terms “ovarian masses,” “adnexal masses,” “tumor markers,” “ultrasound,” “laparoscopy” and “fertility preservation.”

## CASE REPORT

A 21-year-old nulligravid white female presenting with asymptomatic ovarian masses on TVUS was referred for gynecological evaluation. Aside from dyspareunia, the patient was asymptomatic. On ultrasound, the right ovary showed a solid echogenic mass (3.5 x 3.2 x 2.5 cm with a volume of 14 cm^3^) with well-defined limits and smooth walls, suggestive of a dermoid cyst. The left ovary presented with a dense cyst (8.2 x 6.4 x 5.7 cm with 155 cm^3^ volume) with echogenic points and a thin septum. Color Doppler ultrasonography revealed a resistive index (RI) of 0.52.

Pelvic examination showed smooth, cystic, mobile masses occupying both sides of the pelvis. The patient reported no significant past medical history, except for a diagnosis of breast cancer in her mother.

Preoperative investigations included magnetic resonance imaging (MRI), and the levels of tumor markers (alpha fetoprotein [AFP], human chorionic gonadotropin [hCG], lactic dehydrogenase [LDH], CA-125, and cancer antigen 19-9 [CA-19.9]) were assayed. All of these markers were within reference values. MRI revealed a dermoid cyst in the right ovary and a cyst with fine, scarce septa, without nodules or internal vegetations, which could have been compatible with either a functional ovarian cyst or serous cystadenoma. The patient was referred for the surgical treatment of both pelvic masses.

After insertion of a laparoscope, the abdominal and pelvic cavities were carefully assessed with caution. The size of the adnexal masses was determined, and careful inspection of the pelvis was performed to ensure that there was no evidence suggesting malignancy. Bilateral cystectomy was performed by sharp and blunt dissection of the cyst wall from the underlying cortex, followed by intracorporeal ovarian sutures. The preservation of both ovaries was a major surgical goal. An endobag was used for cyst removal. All specimens were sent for pathological examination for definitive pathologic diagnosis. Pathological study of the specimens revealed a cystic teratoma on the right ovary and a mucinous cystadenoma on the left ovary. The patient recovered well and was followed up with TVUS. One year later, the patient returned with a control TVUS showing a dense cyst (2 cm in diameter) with a solid echogenic component (12 mm) on the right ovary. MRI confirmed the presence of a dermoid cyst measuring 2.5 x 3.1 x 1.6 cm. The patient thus underwent another laparoscopy for cystectomy, with preservation of the right ovary. Pathological study was compatible with a dermoid cyst. The patient has been followed since then, with no new recurrences so far.

## DISCUSSION

We aimed at reporting the case of a young woman presenting with simultaneous, distinct ovarian masses treated by laparoscopy with preservation of both gonads and to review the current literature on the subject. Thus, a review of the published literature was conducted by searching Medline and PubMed using the terms “ovarian masses,” “adnexal masses,” “tumor markers,” “ultrasound,” “laparoscopy” and “fertility preservation.”

Incidental adnexal masses represent a wide variety of pathologies, including functional cysts, the sequelae of prior infection, endometriosis, benign or malignant neoplasms, and masses originating from adjacent pelvic organs. TVUS is the preferred modality for initial evaluation in this setting (Liu & Zanotti, 2011).

Adnexal masses may be incidentally detected in an annual pelvic examination, during the work-up of women presenting with symptoms, or as a casual finding on imaging studies performed as part of a diagnostic work-up for an unrelated disease. The majority of such lesions are asymptomatic, unless they rupture or undergo torsion with acute onset of symptoms, such as pelvic pain (Liu & Zanotti 2011; Alcazar *et al*., 2008). As a rule, the diagnostic evaluation of a woman with adnexal mass begins with thorough history taking and physical examination. Imaging and laboratory studies are necessary in most cases.

Histological examination, however, remains the ultimate definitive diagnostic tool (Nezhat *et al*., 2008). In the reproductive age group, the majority of adnexal masses are benign, with malignancy found in only 7-13%.

Functional cysts remain the most common type of adnexal mass found in this age group, and benign cystic teratomas are the most common neoplastic adnexal mass, as reported here (ACOG, 2007; Nezhat *et al*., 2008).

Ultrasound imaging has been shown to be the best diagnostic tool for differentiating malignant from benign adnexal masses, with a sensitivity of approximately 90% and a false-positive rate of approximately 25% (Kinkel *et al*., 2000).

However, the accurate selection of patients with uterine adnexal tumors for surgical intervention is not facilitated by pelvic ultrasonography (Varras, 2004).

For this reason, ultrasound is considered as the first-line imaging technique to be used when assessing an adnexal mass (Alcazar *et al*., 2008; Varras, 2004; Yazbek *et al*., 2007).

Because only the pathology of an adnexal mass can provide a definitive diagnosis, the patient’s age, history, physical examination, and serum marker results, in combination with imaging assessment, such as Doppler sonography, CT, or MRI, should be considered to adequately reach a preoperative diagnosis (Pados *et al*., 2006).

Once an adnexal lesion has been detected, the primary goal of further imaging is accurate tissue characterization, resulting in surgery only for lesions that are indeterminate or frankly malignant.

Lesions that are indeterminate on ultrasound can often be characterized as definitively benign with greater specificity by contrast-enhanced MRI. Anthoulakis *et al*. (2013) conducted a systematic review to critically appraise pelvic MRI as the preferred advanced second-line imaging test for the detection of ovarian cancer and the assessment of indeterminate adnexal masses.

These authors concluded that pelvic MRI should be the method of choice for investigating incidentally discovered, indeterminate, ultrasound-detected adnexal lesions in the general population of post-menarcheal (non-pregnant) women. In the case described here, TVUS was the first exam performed, and MRI was used to confirm diagnosis. As techniques and instruments evolve, laparoscopy and minimally invasive techniques are rapidly emerging as an acceptable alternative to laparotomy for managing adnexal masses and ovarian cancer.

Laparoscopy has the potential to adequately and successfully treat both benign and malignant adnexal pathologies while reducing morbidity among patients. Further advances in technology, techniques, and instruments can only increase this potential (Nezhat *et al*., 2011).

Currently, the laparoscopic management of adnexal masses is the most frequently performed laparoscopic intervention (Pados *et al*., 2006; Liberis *et al*., 2009; Medeiros *et al*., 2009).

The surgical management of benign ovarian tumors must ensure complete removal of the cysts, reduce the risk of recurrence (especially in the case of endometrioma), prevent any risk of tumor dissemination, and preserve healthy ovarian tissue and thus fertility (Borghese *et al*., 2013). Havrilesky *et al*. (2013) evaluated the clinical outcomes of the laparoscopic management of adnexal masses that were thought to be benign preoperatively. Adnexal masses that were thought to be benign preoperatively were successfully managed laparoscopically in three fourths of cases, and the clinical outcomes were acceptable.

Complications were observed in 8% of the cases, and in most cases, adverse events were attributable to concurrent hysterectomy, rather than to surgical treatment of the adnexal mass.

Several studies suggest that ovarian reserve could be reduced after laparoscopic cystectomy due to damage to ovarian vascularity and the removal of an increased amount of ovarian tissue (Li *et al*., 2009; Mohamed *et al*., 2011). However, this reserve may be restored up to 3 months postoperatively in reproductive women (Chang *et al*., 2010).

Others have found that the unwanted effect of bipolar electrocoagulation on ovarian reserve is likely transient and causes minimal transient damage to the ovary. The gentle use of bipolar electrocoagulation or intracorporeal sutures has not been found to affect ovarian reserve (Özgönen *et al*., 2013).

We have been using intracorporeal sutures, as we believe that these sutures have minimal adverse effects on ovarian reserve compared with bipolar electrocoagulation, as measured based on FSH levels (unpublished data).

As TVUS both revealed a cystic teratoma and suggested another benign lesion (mucinous cystadenoma), we decided to perform a laparoscopy as a minimally invasive procedure with preservation of the ovaries.

Mature cystic teratomas (MCTs) are usually asymptomatic and are often discovered incidentally on examination or imaging. The recurrence rate of MCTs following cystectomy is 3-4%, and the incidence of malignant transformation is estimated to be 0.17-2%. Given the accuracy with which MCTs can be diagnosed preoperatively, studies suggest that these lesions can be treated surgically using laparoscopic techniques (O’Neill & Cooper, 2011).

The recommended management of dermoid cysts is generally surgical excision, due to the risk of ovarian torsion, spontaneous rupture, and malignancy. Laparoscopic surgery presents innumerous advantages over laparotomy, such as better visualization of the entire pelvis; reduced analgesia requirements; a shorter hospital stay; prompt recovery, with resumption of activities; and better cosmetic results (O’Neill & Cooper, 2011).

Many reports (Kaminski *et al*., 2006; Kavallaris *et al*., 2010; Briones-Landa *et al*., 2010; Târcoveanu *et al*., 2012; Hursitoglu *et al*., 2013) corroborate that laparoscopic cystectomy of dermoid cysts in premenopausal women is safe and effective and appears to be a valuable alternative to laparotomy.

Controlled intraperitoneal spillage of the cyst contents does not increase postoperative morbidity as long as an endobag is used and the peritoneal cavity is thoroughly washed (Kaminski *et al*., 2006; Kavallaris *et al*., 2010; Briones-Landa *et al*., 2010; Târcoveanu *et al*., 2012; Hursitoglu *et al*., 2013).

Mucinous-type ovarian tumors are the second most common epithelial tumor of the ovary and account for 8-10% of all ovarian tumors. The recurrence of mucinous cystadenomas is said to be very rare after complete excision. However, recurrence may not be as rare as reported in the literature. Intraoperative cyst rupture and cystectomy instead of adnexectomy have emerged as being two risk factors for recurrence (Ben-Ami *et al*., 2010).

Most mucinous ovarian neoplasms (77-87%) are classified as benign. These neoplasms tend to be cystic in nature, and the majority of mucinous tumors (76%) are multilocular, whereas 24% are unilocular (Turkyilmaz *et al*., 2009). In summary, a minimally invasive surgical approach by laparoscopy with preservation of both ovaries is feasible and crucial, even in rare and difficult cases such as the current case.
